# SPIE-AAPM-NCI BreastPathQ Challenge: an image analysis challenge for quantitative tumor cellularity assessment in breast cancer histology images following neoadjuvant treatment

**DOI:** 10.1117/1.JMI.8.3.034501

**Published:** 2021-05-08

**Authors:** Nicholas Petrick, Shazia Akbar, Kenny H. Cha, Sharon Nofech-Mozes, Berkman Sahiner, Marios A. Gavrielides, Jayashree Kalpathy-Cramer, Karen Drukker, Anne L. Martel, for the BreastPathQ Challenge Group

**Affiliations:** a U.S. Food and Drug Administration, Center for Devices and Radiological Health, Silver Spring, Maryland, United States; b University of Toronto, Medical Biophysics, Toronto, Ontario, Canada; c Sunnybrook Health Sciences Centre, Toronto, Ontario, Canada; d University of Toronto, Department of Laboratory Medicine and Pathobiology, Toronto, Ontario, Canada; e Massachusetts General Hospital, Harvard University, Boston, Massachusetts, United States; f University of Chicago, Department of Radiology, Chicago, Illinois, United States

**Keywords:** Grand Challenge, tumor cellularity, machine learning, computer interpretation, prediction probability

## Abstract

**Purpose**: The Breast Pathology Quantitative Biomarkers (BreastPathQ) Challenge was a Grand Challenge organized jointly by the international society for optics and photonics (SPIE), the American Association of Physicists in Medicine (AAPM), the U.S. National Cancer Institute (NCI), and the U.S. Food and Drug Administration (FDA). The task of the BreastPathQ Challenge was computerized estimation of tumor cellularity (TC) in breast cancer histology images following neoadjuvant treatment.

**Approach**: A total of 39 teams developed, validated, and tested their TC estimation algorithms during the challenge. The training, validation, and testing sets consisted of 2394, 185, and 1119 image patches originating from 63, 6, and 27 scanned pathology slides from 33, 4, and 18 patients, respectively. The summary performance metric used for comparing and ranking algorithms was the average prediction probability concordance (PK) using scores from two pathologists as the TC reference standard.

**Results**: Test PK performance ranged from 0.497 to 0.941 across the 100 submitted algorithms. The submitted algorithms generally performed well in estimating TC, with high-performing algorithms obtaining comparable results to the average interrater PK of 0.927 from the two pathologists providing the reference TC scores.

**Conclusions**: The SPIE-AAPM-NCI BreastPathQ Challenge was a success, indicating that artificial intelligence/machine learning algorithms may be able to approach human performance for cellularity assessment and may have some utility in clinical practice for improving efficiency and reducing reader variability. The BreastPathQ Challenge can be accessed on the Grand Challenge website.

## 1 Introduction

Neoadjuvant treatment (NAT) of breast cancer is the administration of therapeutic agents before surgery; it is a treatment option often used for patients with locally advanced breast disease^1^ and more recently is an acceptable option for operable breast cancer of certain molecular subtypes. The administration of NAT can reduce tumor size, allowing patients to become candidates for limited surgical resection or breast-conserving surgery rather than mastectomy.^1^ In addition to affecting parameters, such as histologic architecture, nuclear features, and proliferation,^2^ response to NAT may reduce tumor cellularity (TC), defined as the percentage area of the overall tumor bed comprising tumor cells from invasive or *in situ* carcinoma:^3^

$$\mathrm{TC}=\frac{\text{Tumor bed area containing tumor cells from invasive or}\;in\;situ\;\text{carcinoma}}{\text{Total area of}\;\mathrm{ROI}}.$$

While tumor response to NAT may or may not manifest as a reduction in tumor size, overall TC can be markedly reduced,^4^ making TC an important factor in the assessment of NAT response. TC is also an important component evaluated as part of the residual cancer burden index^5^ that predicts disease recurrence and survival across all breast cancer subtypes.

**Fig. 1:**
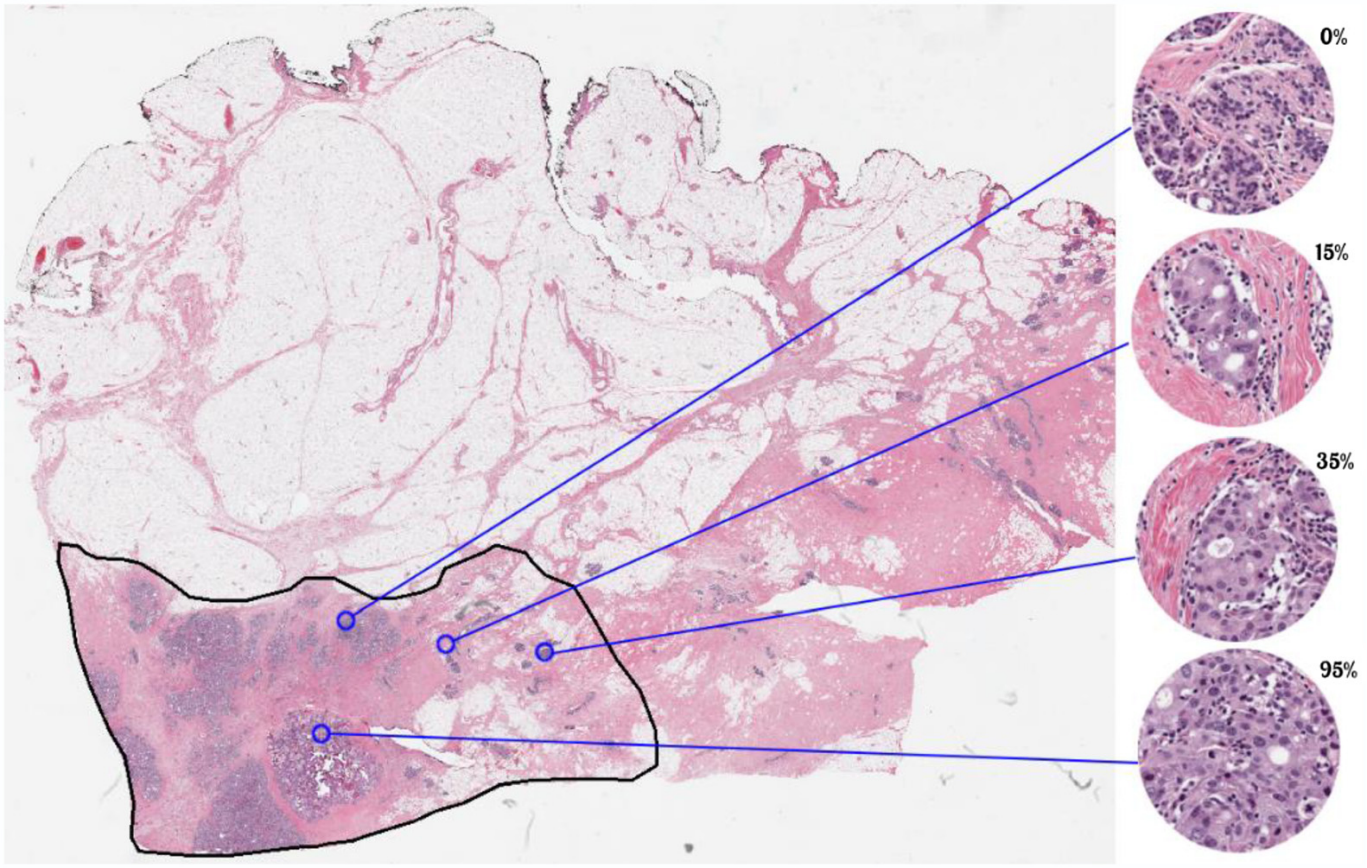
Examples of various levels of TC within different ROIs on an H&E-stained WSI slide.

In current practice, TC is manually estimated by pathologists on hematoxylin and eosin (H&E)-stained slides, a task that is time consuming and prone to human variability. Figure 1 shows examples of various levels of TC within different regions of interest (ROIs) on an H&E stained slide. The majority of practicing pathologists have not been trained to estimate TC as this measurement was only proposed by Symmans et al.^6^ in 2007, and it is currently not part of practice guidelines for reporting on breast cancer resection specimens. That being said, the use of TC scoring is expected to grow because the quantitative measurement of residual cancer burden has proven effective in NAT trials. There is great potential to leverage automated image analysis algorithms for this task to

• provide reproducible and precise quantitative measurements from digital pathology (DP) slides,
• increase throughput by automating part of the tumor burden assessment pipeline, and
• assess TC quickly and efficiently across a large population, which is advantageous in clinical trials.

Digital analysis of pathology slides has a long history dating to the mid-1960’s^7^ with early work by Mendelsohn et al.^8^ analyzing cell morphology from digital scanning cytophotometer images.^9^ More recently, advances in whole slide imaging (WSI) technologies and the recent U. S. Food and Drug Administration (FDA) clearances of the first two WSI systems for primary diagnosis have accelerated efforts to incorporate DP into clinical practice. An important potential benefit of WSI is the possibility of incorporating artificial intelligence/machine learning (AI/ML) methods into the clinical workflow.^10^ Such methods utilize multidimensional connected networks that can progressively develop associations between complex histologic image data and image annotations or patient outcomes, without the need for engineering handcrafted features employed with more traditional machine learning approaches. The potential of AI/ML to improve pathology workflow has been discussed in recent literature.^10^^–^^13^ However, it is challenging to selectively choose the best methods for a given clinical problem because of the vast number of techniques and out-of-the-box models available to algorithm developers, differences between testing datasets, methods for defining a reference standard, and the metrics used for algorithm evaluation.

Global image analysis challenges, such as Cancer Metastases in Lymph Nodes (CAMELYON)^14^ and Breast Cancer Histology (BACH),^15^ have been instrumental in enabling direct comparisons of a range of techniques in computerized pathology slide analysis. Public challenges, in general, in which curated datasets are released to the public in an organized manner, are useful tools for understanding the state of AI/ML for a task because they allow algorithms to be compared using the same data, reference standard, and scoring methods. These challenges can also be useful for improving our understanding of how different choices for reference standards or a different performance metric impact AI/ML algorithm performance and interalgorithm rankings.

This paper describes a challenge directed toward understanding automated TC assessment. The international society for optics and photonics (SPIE), the American Association of Physicists in Medicine (AAPM), the U.S. National Cancer Institute (NCI), and the U.S. Food and Drug Administration (FDA) organized the Breast Pathology Quantitative Biomarkers (BreastPathQ) Grand Challenge to facilitate the development of quantitative biomarkers for the determination of cancer cellularity in breast cancer patients treated with NAT from WSI scans of H&E-stained pathological slides. The Grand Challenge was open to research groups from around the world. The purpose of this paper is to describe the BreastPathQ Challenge design and evaluation methods and to report overall performance results from the Grand Challenge.

## 2 Materials and Methods

### 2.1 Data Acquisition

The dataset for this challenge was collected at the Sunnybrook Health Sciences Centre, Toronto, Canada, following approval from the institutional Ethics Board.^16^ The histopathologic characteristics of the 121 slides from the 64 patients participating in the original study are provided by Peikari et al.^16^ The challenge dataset was a subset of slides from this original study that consisted of 96 WSI scans acquired from tissue glass slides stained with H&E, extracted from 55 patients with residual invasive breast cancer on resection specimens following NAT. Slides were scanned at 20× magnification (0.5  μm/pixel) using an Aperio AT Turbo 1757 scanner (Leica Biosystems Inc., Buffalo Grove, Illinois). Training, validation, and test sets were defined as subsets of the 96 WSI scans: 63 scans (33 patients), 6 scans (4 patients), and 27 scans (18 patients) for the training, validation, and test data sets, respectively. Subsets were defined such that WSI scans from the same patients resided in the same set.

As WSI scans are difficult to annotate due to the sheer volume of data contained within each (between $1\times 10^{9}$ and $3\times 10^{9}\;\text{pixels}$ per WSI scan), we asked a breast pathology fellow (path1) to hand-select patches from each WSI scan, with the intention of capturing representative examples of TC ratings spanning the range between 0% and 100%. This was done using the Sedeen Viewer^17^ (Pathcore, Toronto, Canada). The pathologist drew a small rectangle at the center of the desired patch and then a plugin was used to automatically generate a rectangular ROI of 512×512  pixels around this point. These regions were then passed to an open-source API, Openslide,^18^ to automatically extract 512×512 image patches from the WSI scans, which were then saved as uncompressed TIFF image files. Resulting image files were renamed to reference the WSI scan from which each patch originated. All identifiers were anonymized to maintain patient confidentiality.

**Fig. 2:**
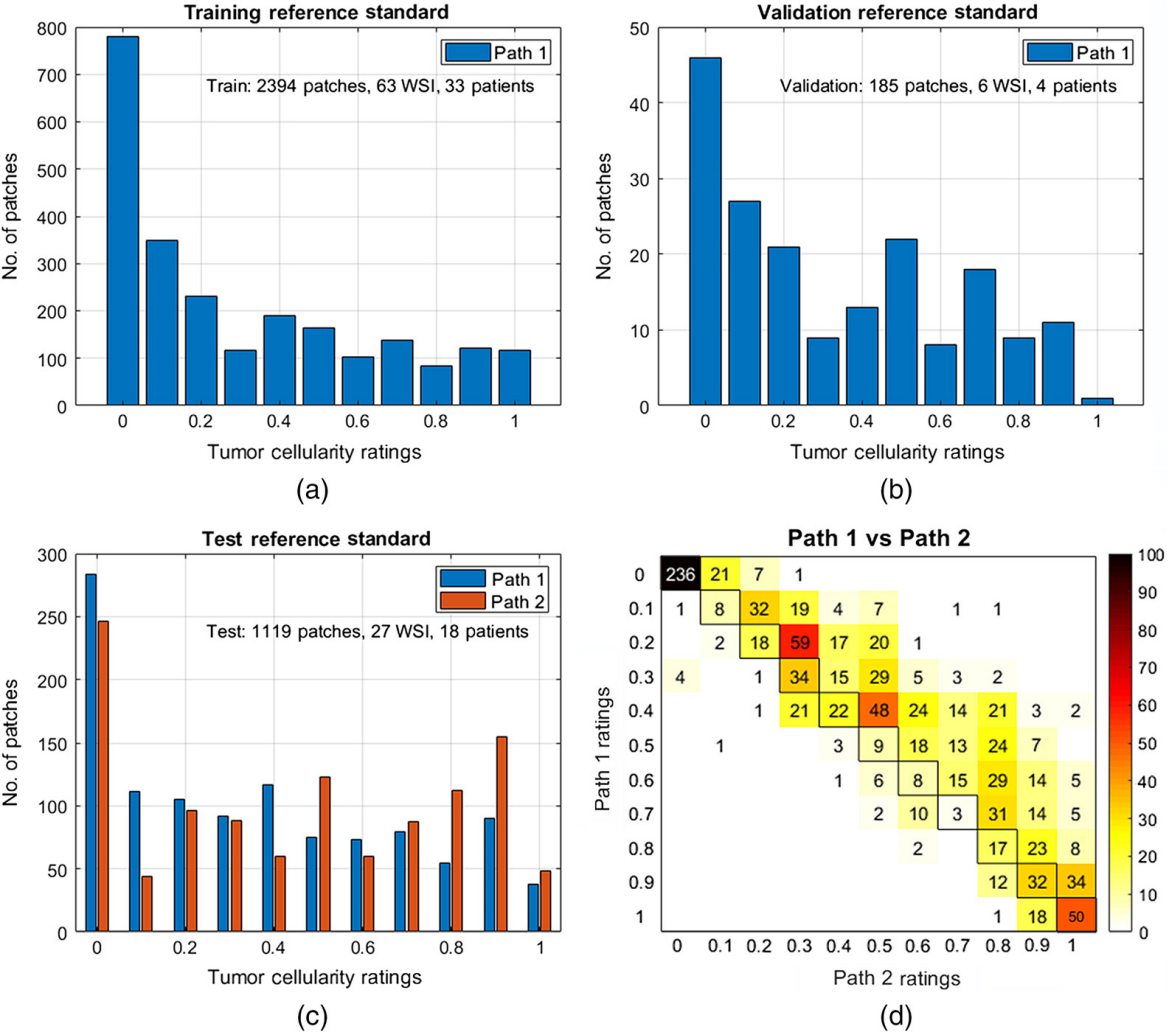
Patch-based histograms of the pathologists’ reference standard scores for the (a) training, (b) validation, and (c) test datasets. (d) Confusion matrix comparing the ratings between path1 and path2 on the test dataset. Only path1 provided ratings for the training and validation datasets, while both path1 and path2 provided ratings for the test dataset.

For each patch, a TC rating, ranging from 0% to 100%, was provided by the pathologist, based on the recommended protocol outlined by Symmans et al.^6^ Patches that did not contain any tumor cells were assigned a TC rating of 0%. The training and validation sets were only annotated by path1, whereas the test set was annotated by both path1 and a breast pathologist (path2). Both path1 and path2 had over 10 years of experience.^16^ Annotations were performed independently, and therefore, each pathologist was unaware of the rating assigned by the other. The distribution of pathologist manual TC ratings used as the reference standard in this challenge for the training, validation, and test sets is given in Fig. 2. The number of patches for which reference standard scores were provided was 2394, 185, and 1119 for training, validation, and test sets, respectively.

Full WSI datasets, in addition to patches, were made available upon request on a password-protected Amazon cloud-based platform, along with instructions for usage of high-resolution DP WSI scans in an image analysis pipeline. Participants were able to request access to the platform via email at the time of the challenge.

### 2.2 Auxiliary Cell Nuclei Dataset

In addition to image patches extracted from WSI scans, participants were also provided with annotations of lymphocytes, malignant epithelial, and normal epithelial cell nuclei in 153 ROIs from the same dataset. These annotations were provided, and participants were permitted to use this in the challenge in addition to the main dataset described above. These data were provided to help developers who wanted to segment cells before calculating a TC score.^16^ In the auxiliary dataset, cell nuclei were marked manually via a pen tool in Sedeen Viewer,^19^ and x-y coordinates were stored in an .xml file for each ROI.

### 2.3 Challenge Setup

The BreastPathQ Challenge was organized with the intention of presenting findings and winners at the BreastPathQ session at SPIE Medical Imaging 2019 (see Sec. 3.2). Participants were allowed to register, and training data were released on October 15, 2018, for the BreastPathQ Challenge. The validation data were released on November 28, 2018, and the test data on December 1, 2018, ∼1 month before the challenge closed on December 28, 2018. Initially holding the validation and test datasets allowed participants time to design their algorithms before assessing their performance. Participants were tasked with assigning TC scores to individual patches during all three phases of the challenge: training, validation, and test. For training purposes, ground truth labels were provided for the training set upon initial release. Subsequently, the ground truth labels for the validation set were released at the time the test patches were released on December 1, 2018. Ground truth for the test set was held out during the entirety of the challenge, only being accessible by the challenge organizers for evaluating the performance of official submissions.

The BreastPathQ utilized an instance of the MedICI Challenge platform to conduct this challenge.^20^ The MedICI Challenge platform supports user and data management, communications, performance evaluation, and leaderboards, among other functions. The platform was used in this challenge as a front-end for challenge information and rules, algorithm performance evaluation, leaderboards, and ongoing communication among participants and organizers through a discussion forum.

The challenge was set up to allow participants to submit patch-based TC scores during the training and validation phases of the challenge and receive prediction probability (PK) performance feedback scores via an automated Python script. The script initially verifies that the submitted score file is valid by checking if the submitted file is formatted correctly and that all patches have a score. An invalid submitted score file was not considered part of the submission limit for the participants. The same evaluation script was used for the training, validation, and test phases. This enabled participants to validate the performance of their algorithms during development as well as familiarize themselves with the submission process prior to the test phase of the challenge.

The submission process involved preparing one TC score per patch in a predefined CSV format described on the website. Participants were also required to provide a description of their submitted method in the form of a two-page algorithm summary as part of the test phase of the challenge. Participants who implemented deep neural networks were asked to provide a description of their network architecture including batch size, optimizer, and out-of-the-box models. Each participant group was allowed up to three valid submissions to be submitted for test set evaluation. Participants were permitted to use additional data in the algorithm development process for pretraining, augmentation, etc., including using their own training data obtained outside the challenge.

### 2.4 Participants

Prior to the test submission deadline on December 28, 2018, there were a total of 317 registrants. During each phase of the challenge, 74, 551, and 100 valid submissions were received for the training, validation, and test phases, respectively. A “valid” submission refers to patch-level predictions successfully submitted by a registered participant. A description of the algorithm(s) was also required as part of a valid test submission. A leaderboard was generated for each phase of the challenge except the test phase; it was updated after each successful submission and was made visible to all participants. The test leaderboard results were hidden from the participants. Results of the challenge were announced at a special SPIE BreastPathQ session that took place during a joint session with the 2019 SPIE Medical Imaging Computer Aided Diagnosis conference and Digital Pathology conference in San Diego, California, held from February 16 to 21, 2019. During the session, the top two winners presented their algorithm and performance in oral presentations. Other participants were also invited to present their methods in a poster session during the conference.

A list of the 39 teams who submitted valid test set entries is provided in Appendix A with teams being allowed to submit up to three algorithms in the test phase. Members of the organizing committee, as well as students and staff from their respective organizations, were not permitted to participate in the challenge due to potential conflicts of interest.

### 2.5 Evaluation Metric

The primary evaluation metric used for determining algorithm rankings and the winners for the challenge was PK. Intraclass correlation analysis was also performed as a secondary analysis, unknown to the challenge participants, to compare with the PK rankings and results. As two pathologists provided reference standard TC scores for the test set, the PK results based on each individual pathologist were averaged to get a final average PK for each algorithm. The 95% confidence limits [upper and lower bounds (UB and LB, respectively)] of each summary performance score were calculated using bootstrapping (resampling with replacement) 1000 times on a per-patient basis and obtaining the 95% confidence interval using the percentile method.

#### 2.5.1 Prediction probability score

PK^21^ is a concordance metric that measures the agreement in the ranking of paired cases by two readers or algorithms. It was used as the main evaluation metric for the challenge specifically because it was not clear if the two pathologist TC predictions would be well calibrated due to interpathologist variability. Concordance evaluates the ranking of cases but not the absolute values such that calibration between readers or between a reader and algorithm is not required. Patch ranking was deemed the most important comparison to assess since calibrating an algorithm could potentially be achieved as an additional step for well-performing algorithms. PK is defined as

$$P_{k}=\frac{1}{2}(\frac{C-D}{C+D+T_{A}}+1)=\frac{C+\frac{1}{2}T_{A}}{C+D+T_{A}},$$

where C is the number of concordant pairs, D is the number of discordant pairs, and $T_{A}$ is the number of ties in the submitted algorithm results. PK can be defined as the probability that the method ranks two randomly chosen cases in the same order as the reference standard. It is also a generalization of the trapezoidal area under the receiver operating characteristics curve (AUC) calculation. The PK was calculated by modifying SciPy’s^22^ implementation of Kendall’s Tau-b. SciPy’s implementation calculates the components (C, D, $T_{A}$) needed for the PK estimation such that our modification simply involved using the estimated components to calculate PK. The Python function for calculating PK was made available to all participants of the challenge.

#### 2.5.2 Intraclass correlation value

Concordance measures the similarity between the rankings of patches by two readers/algorithms, but it does not require calibration of the algorithm and the reference TC scores. After reviewing the various deep learning algorithm implementations, it was clear that mean squared error (MSE), a correlation measure between an algorithm’s TC outputs and the reference standard values, was commonly used to optimize algorithm performance. Calibration differences between the algorithm and the references do impact MSE. Since MSE was such a common optimization metric, we added a secondary correlation analysis as part of the challenge analysis plan, namely the intraclass correlation coefficient (ICC), to better understand the impact of the performance metric on algorithm rankings. The ICC was calculated using two-way effects with absolute agreement [ICC (2,1) by the Shrout and Fleiss convention^23^], using the “irr” package^24^ in R.

### 2.6 Patch-Based Mean Squared Error Analysis

Another *post hoc* analysis performed after completion of the challenge was the calculation of the patch-based average MSE between the pathologists and all submitted algorithms to identify which patches had the largest and the smallest errors in predicting TC. The MSE between each pathologist and the algorithms for an individual patch was calculated as the squared sum across all algorithms of the difference between the pathologist TC score and an individual algorithm TC prediction. The final MSE value was then the average across the two pathologists. A higher MSE indicated that the algorithms performed relatively poorly in predicting the cellularity for a patch, whereas a lower MSE indicated better performance.

## 3 Results

### 3.1 Submitted Algorithms

The BreastPathQ Challenge participants represented a total of 39 unique teams from 12 countries. Almost all of the teams (38/39) used deep convolutional neural networks (CNNs) to build their automated pipelines, with most also using well-established architectural designs (Sec. 3.1.2). The participants also universally employed data augmentation techniques (Sec. 3.1.1) to enhance algorithm training and performance. The remainder of this section summarizes various aspects of the submitted algorithms in more detail, and a brief summary of all submitted methods is provided in Appendix A.

#### 3.1.1 Preprocessing/data augmentation

All participants used some form of data augmentation to increase the size of the original dataset, with most of the participants employing random rotations, flips, and color jittering. Some participants also opted to use the HSV (hue–saturation–value) color space in addition to, or in combination with, the RGB (red-green-blue) color space.

#### 3.1.2 Neural network architectures

The top 10 performing teams in the BreastPathQ Challenge used deep neural networks to generate TC scores, and they all used pretrained CNN architectures, including Inception^25^, ResNet,^26^ and DenseNet.^27^ Other commonly used CNN architectures included Xception,^28^ VGG,^29^ and SENet.^30^ Other teams developed custom networks. Ensembles of deep learning-based networks were also a common approach for achieving improved algorithm performance. The two top performing teams incorporated squeeze-and-excitation (SE) blocks^30^ in their pretrained Inception and ResNet models. SE blocks integrate into existing network architectures by learning global properties along with traditional convolutional layers. The SE block itself captures global properties in a network by aggregating feature maps along their spatial dimensions followed by a “self-gating mechanism.”^30^

Typically, CNN outputs were linearly mapped to scores between 0 and 1, and distance-based loss functions were adopted to perform backpropagation. The most commonly used loss function was MSE; however, other common losses such as least absolute deviation (L1) were also used.

The majority of CNNs (except custom-made CNN architectures) used ImageNet^31^ pretrained weights. Public datasets were also used for pretraining, including the BACH challenge dataset, which includes H&E-stained breast histology microscopy and WSI scans representative of four types of breast cancer.^15^ One participant also used the large 2018 data science bowl challenge dataset of cell nuclei from various types of microscopic imaging modalities.^32^ Aside from CNNs, two participants used unlabeled data in the hopes of avoiding overfitting in the task. Team ThisShouldBeOptional pretrained a generative adversarial network (GAN)^33^ with data from the 2014 International Conference on Pattern Recognition (ICPR) contest^34^ and then trained on the BreastPathQ dataset, using the discriminator to predict TC scores. Team max0r similarly used the discriminator of an InceptionNet^35^ adversarial autoencoder to regularize the feature space prior to training for prediction of TC scores.

#### 3.1.3 Cell segmentation

The auxiliary dataset described in Sec. 2.2 was adopted by various participants to incorporate cell segmentation and classification as tumor versus normal in their pipelines. Because the cell nuclei locations were given as x-y coordinates, some participants chose to sample patches centered at the provided coordinates while others simulated segmentation maps by drawing circles around these points (e.g., Team rakhlin). There was a range of different architectures used to perform TC score prediction from cell segmentation maps including U-Net,^36^ fully connected networks (FCN),^37^ and custom network designs.

#### 3.1.4 Postprocessing

We found that all participants who used CNNs also employed some sort of ensemble method. Most opted to use k-fold cross-validation to split the training set and learn individual models per fold. Final TC scores were achieved mostly through either an averaging/maximum operation or learning a separate regression layer that aggregated penultimate layers in each CNN. Some participants also trained individual CNNs with different architectures in parallel and combined results using one of the above methods.

Due to the nature of the task, and because scores were discretized through manual assessment, two participants performed a combination of classification and regression. Team SCI performed classification by artificially creating multiple classification categories, whereas Team SRIBD opted to learn a label distribution automatically via label distribution learning.^38^ Training was then performed on a combination of two (or more via ensemble) sets of ground truth labels.

### 3.2 Prediction Probability Analysis

The best performing method on the independent test set achieved an average PK of 0.941 [0.917,0.958], which was comparable to but also slightly higher than the average interrater PK of 0.927 [0.914,0.940] for path1 and path2, who provided the reference standard TC scores for the dataset. The PK of the best-performing algorithm failed to reach a statistically significant difference from that of the individual pathologists’ PKs.

**Fig. 3:**
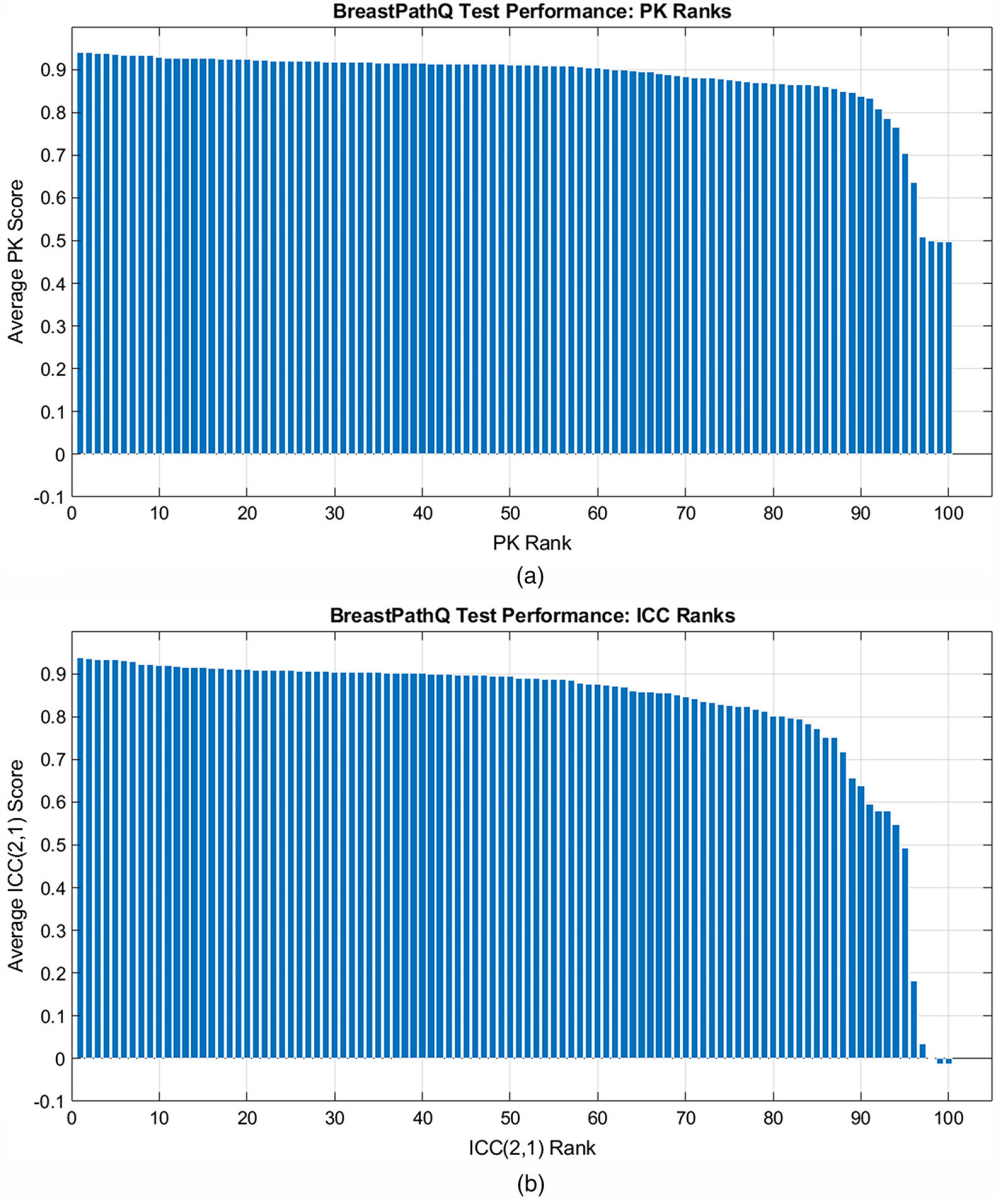
Average scores sorted by the participants’ ranks for (a) test PK scores and (b) test ICC (2,1) scores.

**Fig. 4:**
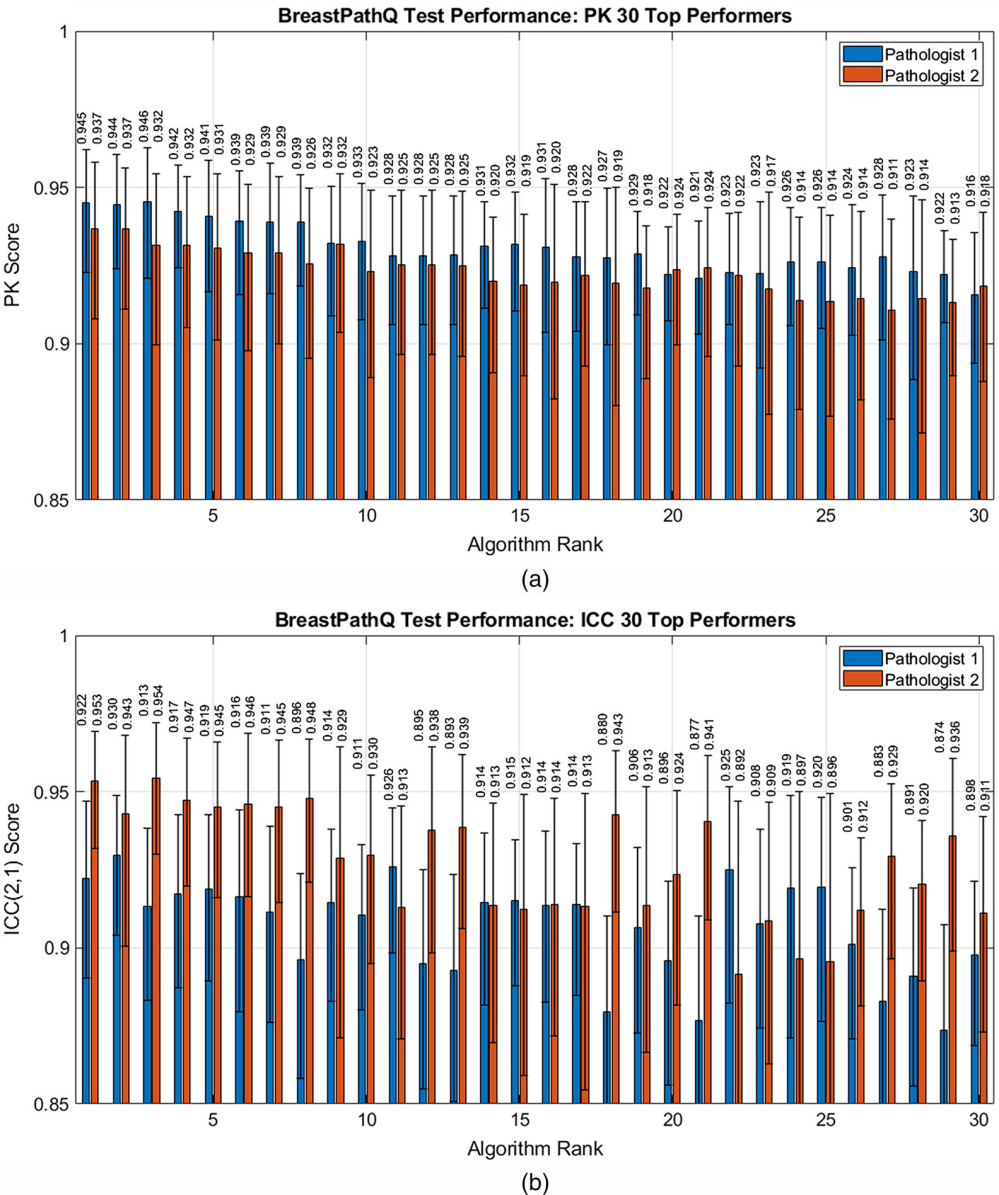
Algorithm scores using the individual pathologists as the reference standard sorted by the participants’ ranks for the top 30 performers. (a) Test PK scores by top average PK scores and (b) test ICC (2,1) scores by top average ICC scores.

Figure 3(a) shows the average PK scores sorted by algorithm from highest to lowest rank with the actual PK scores given in Table 1. Figure 4(a) shows the individual PK scores using either path1 or path2 as the reference standard for the top 30 performing algorithms in terms of average PK score. PK was generally higher for path1 as the reference as opposed to path2 for this set of high-performing algorithms.

**Table 1:** Best PK scores and the corresponding ICC values achieved by each BreastPathQ participant team, averaged between two pathologists. Some ranks (e.g., rank 5) are not listed because a different algorithm from the same team achieved a higher rank.

Rank	PK score			ICC score		
	Average	Lower Bounds	Upper Bounds	Average	Lower Bounds	Upper Bounds
1	**0.941**	0.917	0.958	**0.934**	0.908	0.954
2	**0.941**	0.920	0.957	**0.938**	0.913	0.956
3	**0.939**	0.911	0.958	**0.936**	0.906	0.957
4	**0.937**	0.916	0.955	**0.922**	0.892	0.943
6	**0.934**	0.906	0.953	**0.928**	0.896	0.952
8	**0.932**	0.908	0.952	**0.911**	0.879	0.934
9	**0.932**	0.908	0.951	**0.931**	0.901	0.954
11	**0.927**	0.908	0.947	**0.861**	0.807	0.901
14	**0.926**	0.902	0.942	**0.895**	0.852	0.924
18	**0.923**	0.890	0.950	**0.908**	0.845	0.949
20	**0.923**	0.904	0.938	**0.904**	0.874	0.930
26	**0.919**	0.893	0.943	**0.910**	0.873	0.939
27	**0.919**	0.890	0.943	**0.885**	0.835	0.918
29	**0.918**	0.899	0.934	**0.902**	0.870	0.926
30	**0.917**	0.891	0.939	**0.900**	0.859	0.930
31	**0.917**	0.887	0.940	**0.902**	0.863	0.929
33	**0.916**	0.895	0.933	**0.906**	0.877	0.928
36	**0.916**	0.896	0.932	**0.855**	0.810	0.888
37	**0.915**	0.897	0.932	**0.904**	0.880	0.926
38	**0.915**	0.890	0.936	**0.904**	0.874	0.929
40	**0.913**	0.893	0.928	**0.906**	0.874	0.929
41	**0.913**	0.892	0.931	**0.914**	0.884	0.937
45	**0.912**	0.892	0.929	**0.903**	0.872	0.928
53	**0.907**	0.861	0.939	**0.890**	0.821	0.934
55	**0.904**	0.872	0.930	**0.895**	0.836	0.931
56	**0.902**	0.877	0.930	**0.876**	0.839	0.906
57	**0.900**	0.879	0.921	**0.888**	0.851	0.919
66	**0.880**	0.838	0.910	**0.579**	0.540	0.611
67	**0.879**	0.856	0.900	**0.858**	0.810	0.902
68	**0.876**	0.848	0.899	**0.859**	0.802	0.897
70	**0.870**	0.843	0.889	**0.828**	0.779	0.865
71	**0.869**	0.813	0.913	**0.812**	0.689	0.898
72	**0.868**	0.826	0.902	**0.837**	0.759	0.886
73	**0.866**	0.845	0.888	**0.843**	0.789	0.885
75	**0.864**	0.837	0.889	**0.824**	0.780	0.871
78	**0.860**	0.777	0.908	**0.772**	0.579	0.884
80	**0.847**	0.790	0.900	**0.801**	0.709	0.872
84	**0.785**	0.711	0.864	**0.656**	0.497	0.796
87	**0.497**	0.476	0.517	**−0.012**	−0.054	0.041

**Fig. 5:**
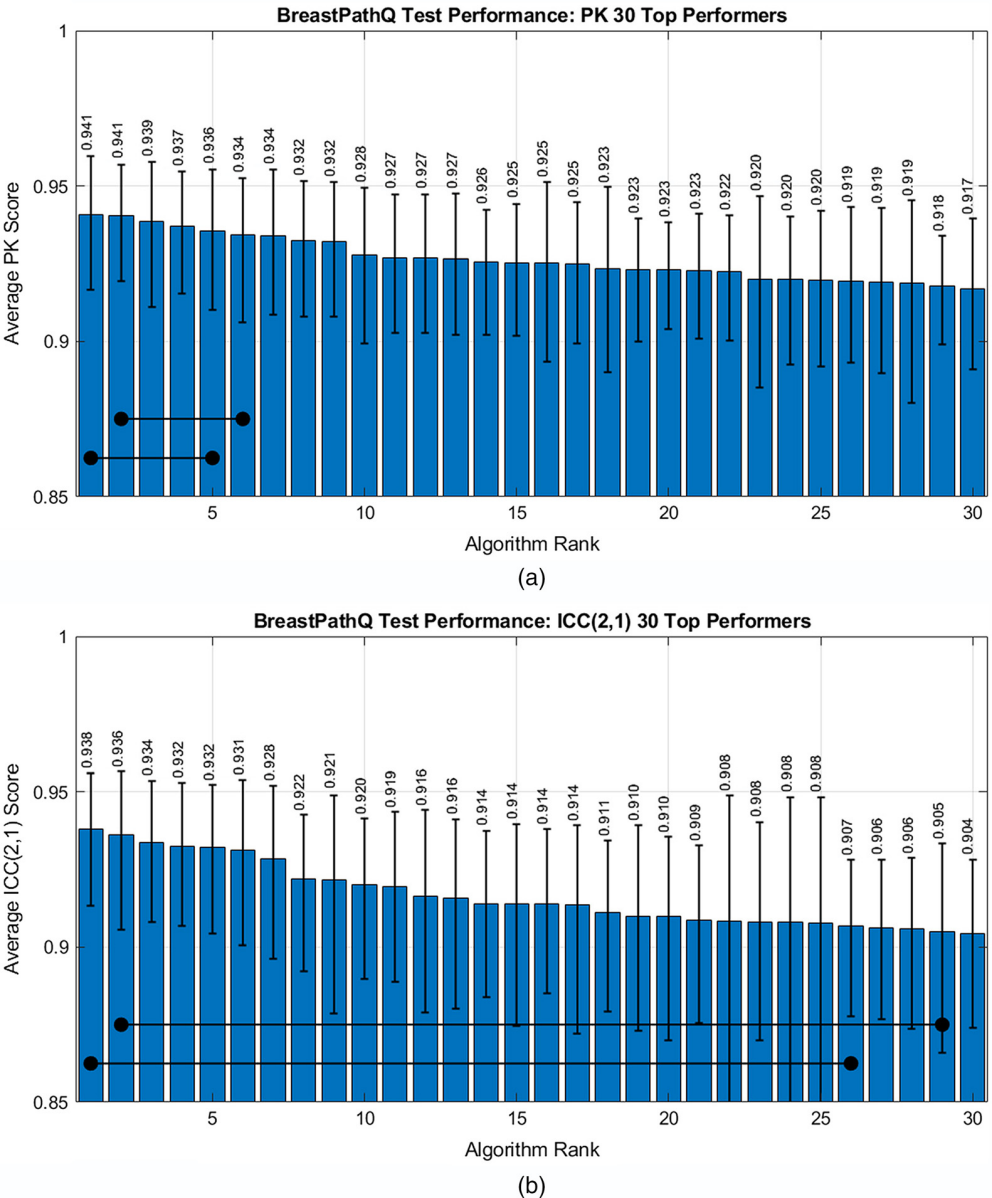
Average performance sorted by participants’ ranks for the top 30 performers in (a) test PK scores and (b) test ICC (2,1) scores. The horizontal lines show the first algorithm in which there is a statistically significant difference between the two bullets.

Figure 5(a) focuses on the average PK for the top 30 performers and shows a relatively small range in performance of 0.917 to 0.941 across the 30 algorithms. The figure also indicates the first algorithm with PK performance that is statistically significantly different from the first- and the second-ranked algorithms at the α=0.05 level. The first ranked algorithm (PK=0.941 [0.917,0.958]) was statistically superior to the fifth ranked algorithm (PK=0.936 [0.910,0.955]) and all subsequent lower ranked algorithms. The second ranked algorithm (PK=0.941 [0.920,0.957]) was statistically superior to the sixth ranked algorithm (PK=0.934 [0.906,0.953]) such that a difference of about 0.006 in PK was statistically significant for the top performing algorithms.

### 3.3 Intraclass Correlation Analysis

ICC values were not an endpoint of the BreastPathQ Challenge in that these results were not used to select the challenge winners; however, we decided to compute and report ICC values after completion of the competition to determine the impact of algorithm ranking on the use of either a rank-based or a calibrated endpoint. The best-performing method achieved an average ICC of 0.938 [0.913,0.956], which was higher than the average inter-rater ICC of 0.892 [0.866,0.914] between path1 and path2.

Figure 3(b) shows the average ICC scores sorted by algorithm from highest to lowest rank with the best ICC score by participant given in Table 1. Figure 4(b) shows ICC scores using path1 or path2 as the reference standard for the top 30 performing algorithms in terms of average ICC score. In this case, the ICC was generally higher for path2 as the reference compared with path1 as the reference. This trend is the reverse of what was observed for the highest performing PK algorithms in which comparisons with path1 typically resulted in higher PK.

**Fig. 6:**
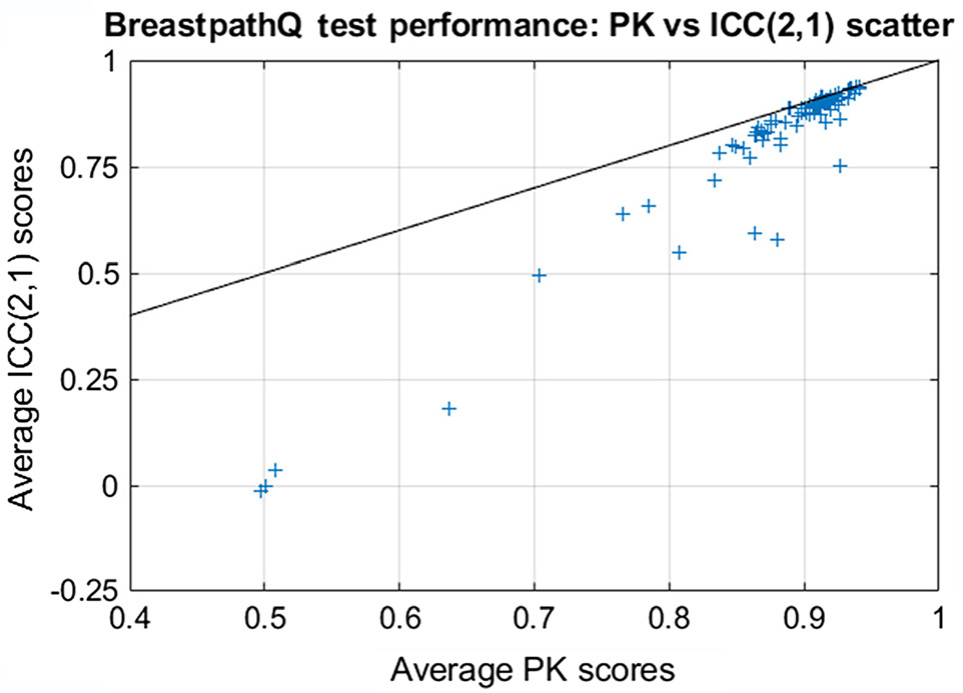
Scatter plot between average PK scores and average ICC (2,1) scores. The solid back curve is line with slope=1.

Figure 5(b) focuses on the average ICC for the top 30 performers. The range in average ICC was 0.904 to 0.938 across the 30 algorithms. The figure also shows that the first ranked ICC algorithm (ICC=0.938 [0.913,0.956]) was statistically superior to the 26th ranked algorithm (ICC=0.907 [0.878,0.928]) and all subsequent lower ranked algorithms. The 2nd ranked algorithm (ICC=0.936 [0.906,0.957]) was statistically superior to the 29th ranked algorithm (ICC=0.905 [0.866,0.933]) such that a difference of about 0.031 in the ICC was statistically significant for the top performing algorithms. This ICC difference for statistical significance was substantially larger than the ∼0.006 needed for PK significance. However, looking at the scatter plot of PK scores versus ICC scores in Fig. 6, we see that the ranks in the two reference standard approaches were fairly consistent in that high performers in PK tended to be high performers in ICC as well.

### 3.4 Patch-Based Analysis

**Fig. 7:**
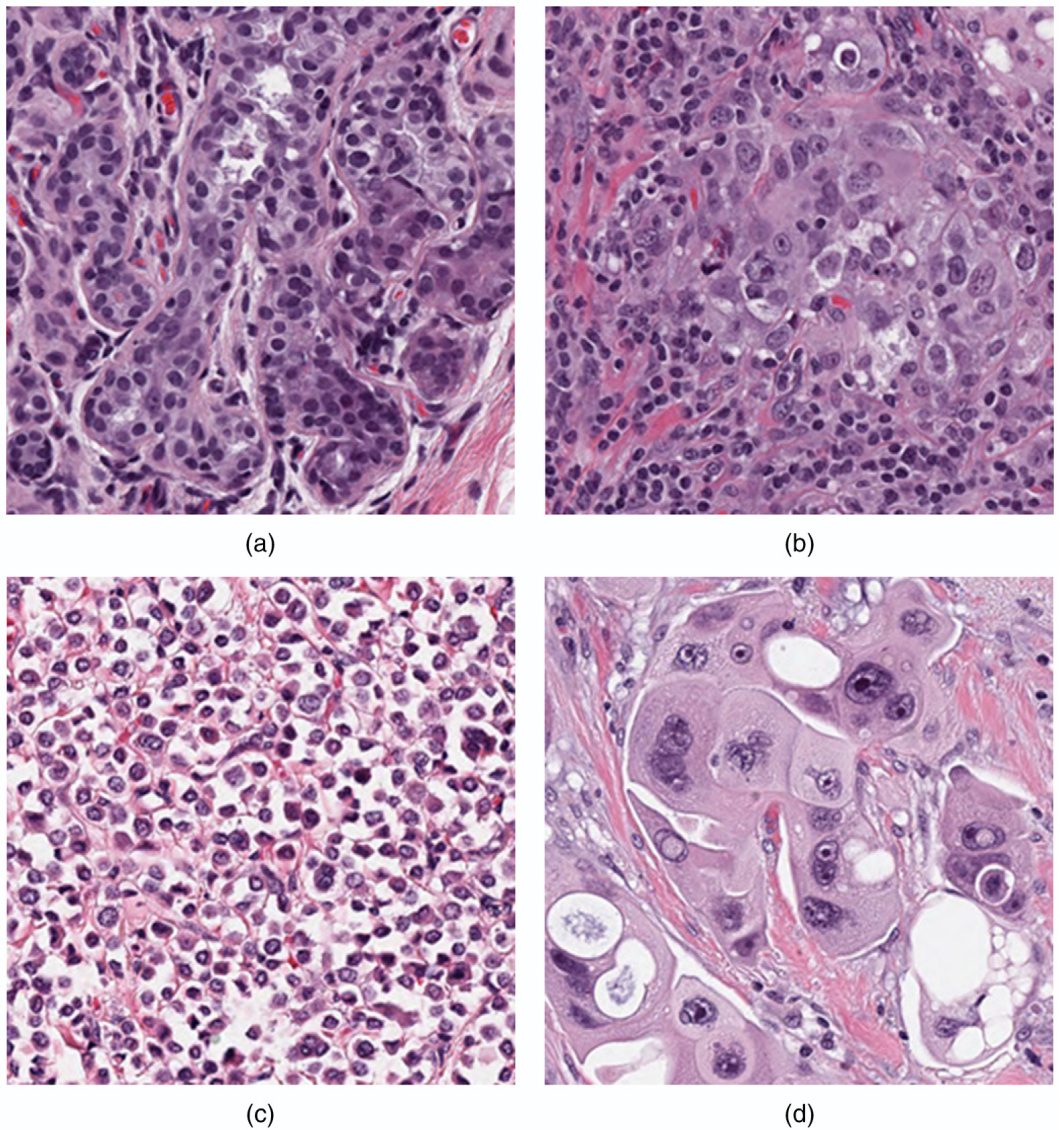
Patches with the high average MSE between the pathologists and the algorithms. The MSE, path1 TC score, path2 TC score, and average algorithm TC score for each patch (a)–(d) is given below. (a) MSE=0.364, path1=0, path2=0, AvgScore=0.53±0.28, score range=[0,0.98], (b) MSE=0.247, path1=0.35, path2=0.3, AvgScore=0.78±0.18, score range=[0.10,1.00], (c) MSE=0.191, path1=0.9, path2=1, AvgScore=0.58±0.23, score range=[0,0.99], and (d) MSE=0.093, path1=0.8, path2=0.8, AvgScore=0.52±0.11, score range=[0.24,0.82].

**Fig. 8:**
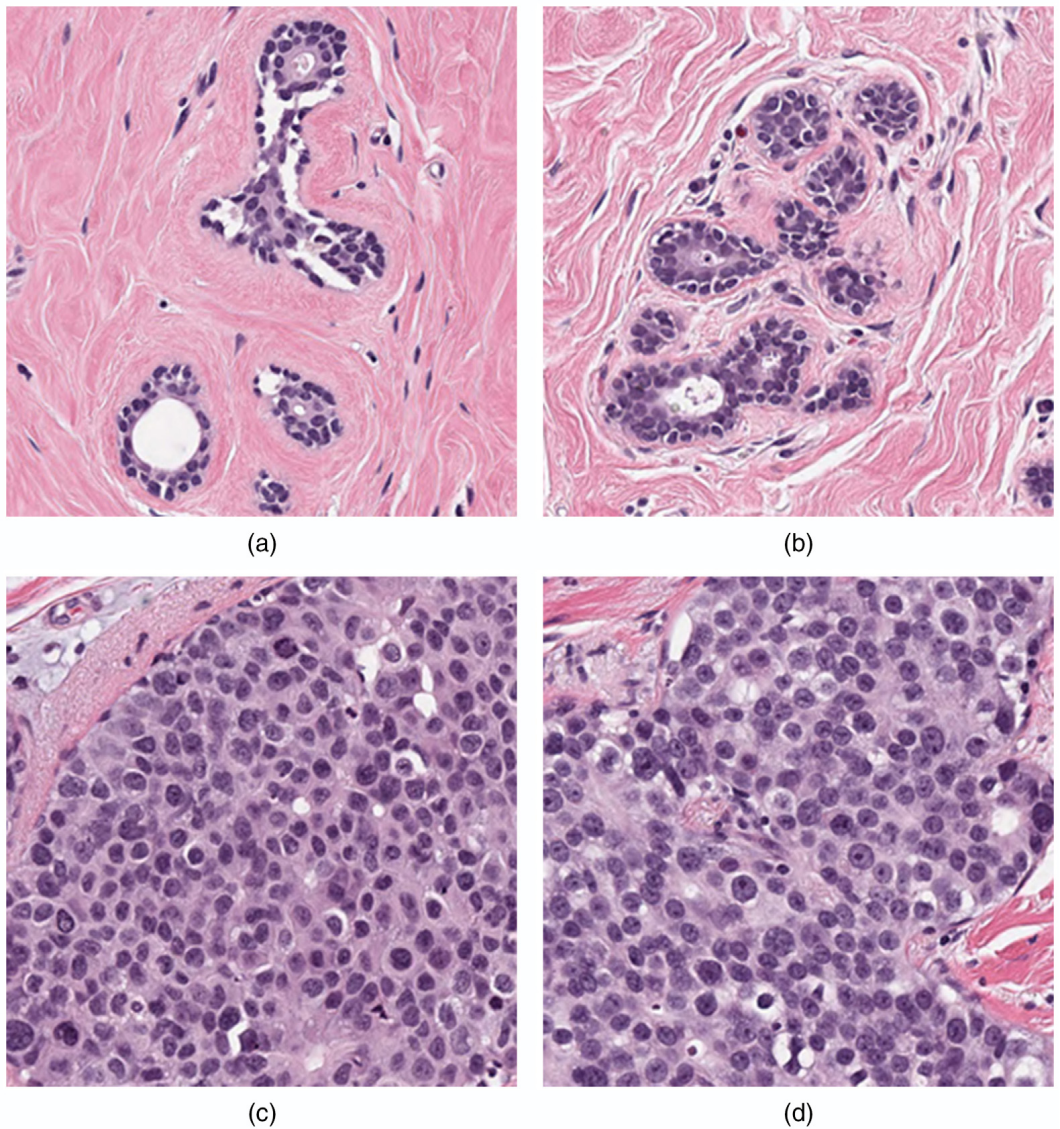
Patches with the lowest average MSE between the pathologists and the algorithms. The MSE, path1 TC score, path2 TC score, and average algorithm TC score for each patch (a)–(d) is given below. (a) MSE=0.004, path1=0, path2=0, AvgScore=0.03±0.06, scorerange=[0,0.30], (b) MSE=0.006, path1=0, path2=0, AvgScore=0.03±0.07, scorerange=[0,0.50], (c) MSE=0.011, path1=0.95, path2=0.9, AvgScore=0.90±0.10, score range=[0.41,1.00], and (d) MSE=0.0133, path1=0.9, path2=0.9, AvgScore=0.88±0.11, score range=[0.40,1.00].

Figures 7 and 8 show patches in which the patch-based MSE were the highest and the lowest, respectively, along with the average algorithm TC score (AvgScore). The algorithms performed poorly for the examples shown in Fig. 7, by overestimating TC for the region of closely packed benign acini seen in the sclerosing adenosis of Fig. 7(a) and in the patch depicting a high number of tumors associated with inflammatory cells in Fig. 7(b). The algorithms underestimated TC for the lobular carcinoma in Fig. 7(c), which is characterized by sheets of noncohesive cells with nuclei only slightly larger than inflammatory cells that do not form tubules or solid clusters. TC was also consistently underestimated in the apocrine carcinoma depicted in Fig. 7(d), which had markedly abundant cytoplasm such that the surface area of the tumor cells is significantly larger than the surface area of the nuclei.

On the other hand, the algorithms performed quite well for the patches that depicted benign, completely normal breast lobules in the acellular stroma shown in Figs. 8(a) and 8(b) and in acellular stroma and for malignant patches in Figs. 8(c) and 8(d) showing cohesive residual tumor cells with high nuclear–cytoplasmic ratio encompassing the majority of the surface area. In these cases, the tumor–stroma interface was well delineated, and the stroma contained a minimal number of inflammatory cells.

## 4 Discussion

The submitted algorithms generally performed quite well in assessing cancer cellularity for H&E breast cancer tumor patches with the majority, 62/100 submitted algorithms, having a PK scores greater than 0.90 on a scale of 0.0 to 1.0. The top performing algorithms had PK comparable to path1 and path2 pathologists who had an average interrater PK of 0.927 [0.914,0.940] on the test dataset. This indicates that a range of different deep learning approaches (e.g., ResNet50, squeeze-excitation Resnet50, DenseNet, Xception, Inception, and ensembles of architectures) may be able to perform similarly to pathologists in ranking pairs of slide patches in terms of cellularity. A similar trend was observed with the ICC metric in which 50/100 algorithms had mean ICC performance above 0.892, the average interrater ICC performance on the test dataset. This ICC performance again suggests that a range of deep learning techniques can produce similar cellularity scores to those of the pathologists participating in this study such that automated cancer cellularity scoring may be a reasonable AI/ML application to consider. The value of a successful AI/ML implementation could be in streamlining the assessment of residual cancer burden in breast and other cancers and reducing the variability in cellularity scoring compared with that of pathologists. While the challenge results are encouraging, this is an early stage study that simply indicates that some of the better performing algorithms may have merit for further optimization and testing. Algorithm performance would need to be confirmed on a much larger and more diverse dataset to verify both the algorithm performance and consistency with pathologist interpretation across different patch types. Such a dataset should consist of images acquired with different scanners and acquired at different sites so that it would be representative of the image quality observed in clinical practice. This challenge included only images scanned using a single WSI scanner and from a single site. In addition, our reference standard was limited to two pathologists, and these pathologists exhibited variability in their TC scores, indicating that a larger study should include a larger, more representative group of pathologist readers to better account for reader variability.

While overall performance was good for the top performing algorithms, it was observed that AI/ML algorithms as an entire group tended to perform well or poorly for some patches. Figures 7 and 8 show some insight into the errors made by the algorithms. Figure 8 shows examples of “typical” appearing patches where the algorithms tend to do well, in terms of low average MSE with the pathologists. The zero cellularity patches in Figs. 8(a) and 8(b) show the classical appearance of normal breast tissue where epithelial cells form ducts and are surrounded by regions of stroma, while the high cellularity patches in Figs. 8(c) and 8(d) contain dense regions of randomly arranged malignant epithelial cells. The patches in Fig. 7 cause the most difficulty for the submitted algorithms in general; Fig. 7(a) shows cellularity in a region derived from a patient with adenosis. While this is benign, the dense concentration of epithelial cells seems to have been mistaken for cancer by many of the algorithms, leading to high TC scores compared with the pathologists scores. Similarly, the high concentration of tumor infiltrating lymphocytes in Fig. 7(b) led to an overestimation of cellularity by the algorithms. In Fig. 7(c), the tumor cells in the lobular carcinoma are distorted and noncohesive, while the effect of the NAT led to a high cytoplasm to nuclei ratio, which caused the algorithms to underestimate cellularity in Fig. 7(d). These figures suggest that the challenge algorithms, as a group, performed relatively well on easier patches (Fig. 8) and struggled on more difficult patches (Fig. 7) in which pathologists may benefit most from an AI/ML. The errors also demonstrate the degree of variability in tumor cell properties across breast cancer cases treated with NAT and demonstrate that large and representative datasets are needed to train and evaluate models for DP implementation. Algorithm evaluation with large datasets can also serve to document the types of cases in which AI/ML performs well and those types that are problematic.

Ensemble methods, which combine the output of multiple trained neural networks into a single output, have become a common approach for challenge participants for improving AI/ML algorithm performance. It was the same for the BreastPathQ Challenge, in which most of the teams used an ensemble of deep learning algorithms instead of limiting themselves to just a single deep learning architecture and training. In general, the ensemble method had higher PK performance than the nonensemble methods, and the top five algorithms in terms of PK all used an ensemble of deep learning architectures. The advantage of ensembles or combinations of algorithms leading to improved performance was also observed in the DM DREAM Challenge, in which the ensemble method significantly improved the AUC over the best single method from 0.858 to 0.895^39^ for the binary task of cancer/no cancer presence in screening mammography. Our results indicate that ensembles of deep-learning architectures can improve estimation performance in independent testing compared with single classifier implementations at the cost of additional training time and validating the multiple neural networks.

Our initial choice for concordance metric was Kendall’s Tau-b ($\tau_{B}$). $\tau_{B}$ is a common metric for concordance^40^ and is given as

$$\tau_{B}=\frac{C-D}{\sqrt{\left(C+D+T_{A})*(C+D+T_{R}\right)}},$$

where C is the number of concordant pairs, D is the number of discordant pairs, $T_{A}$ is the number of ties in the submitted algorithm results, and $T_{R}$ is the number of ties in the reference standard. However, one of the participants in the challenge (David Chambers, Southwest Research Institute, Team: dchambers) identified a problem with $\tau_{B}$ early after the initial release of the training data. The participant found, and we confirmed through simulations, that by simply binning continuous AI/ML algorithm outputs (e.g., binning scores to 10 equally spaced bins between 0 and 1 instead of using a continuous estimate between 0 and 1) one could artificially increase the number of ties $T_{A}$ that an algorithm produces. Binning also impacted the number of concordant C and discordant D pairs. Based on our simulation studies, we found that binning decreased the number of concordant pairs C somewhat but also lead to a much larger decrease in the number of discordant pairs D because regions having similar TC scores are more difficult to differentiate than regions having large differences in TC in general. Binning had a relatively small impact on the $\tau_{B}$ denominator such that the overall effect was to increase $\tau_{B}$ compared with using continuous TC estimates or even smaller bin sizes. To prevent the possibility of the challenge results being manipulated through the binning of algorithm outputs, we revised our initial concordance endpoint to use the PK metric, which does not suffer from this shortcoming. Increasing algorithm ties $T_{A}$ by binning still impacts C and D, but the large reduction in D reduced the PK denominator $C+D+T_{A}$ to a larger degree than the numerator $C+\frac{1}{2}T_{A}$ such that binning algorithm estimates tend to reduce PK instead of improving it.

As described in Sec. 2.1, path1 provided all of the reference label scores for the training and validation data. Figure 4(a) shows that test PK performance for an algorithm was consistently larger having path1 as the reference standard compared with path2 for almost all top 30 performers, although the error bars largely overlap. One possible explanation for this consistent difference is that the participants may have been able to tune their algorithms to path1 TC scores during the training and validation phases since path1 was the reference label for these datasets. Although PK was not explicitly used as part of the loss function for algorithm training by any participants, it is likely that they selectively submitted algorithms during the test phase that produced higher PK performance in the training and validation phases. It is not surprising to see better PK performance for path1 compared with path2 since path1 was the reference labeler for all three datasets.

Interestingly, the trend was opposite for the ICC. Figure 4(b) shows algorithm ICC performance for both reference labelers on the test dataset. The ICC with path2 as the reference are larger than the ICC with path1 as the reference for most of the top ICC preforming algorithms. Participants did not optimize their algorithm for the ICC nor did they receive feedback on ICC performance during the course of the challenge. In addition, when using path 2 as the reference, the difference in the ICC between the algorithms and path1 was statistically significant but not vice-versa. We hypothesize that this is likely a coincidence in our study due to having two different truthing pathologists and no algorithm optimization toward the ICC endpoint. For PK, the difference between the algorithms and the individual pathologists failed to reach statistical significance. This result suggests that ICC performance, in which calibration in the scores is accounted for, performs differently than PK, a rank-based performance metric. Despite this, many of the top performing PK algorithms were also among the top ICC performers. This can be seen by studying Fig. 4 where the top three algorithms in terms of PK are also the top three in terms of ICC. Likewise, 8 of the top 10 performing PK performers are in the top 10 performing ICC algorithms. We conjecture that if the challenge had returned ICC performance to the participants in the training/validation stage instead of PK, Fig. 4(b) would likely have shown better ICC performance for path1 over path2 because the participants would have adjusted their submissions to those with higher ICCs on the training and validation datasets. Therefore, we believe it is important to consider what performance feedback is provided to participants in an AI/ML challenge since this can impact which models are submitted. The results indicate a limitation of the challenge of having only a single pathologist provide a reference TC score for the training and validations datasets. This suggests that it is reasonable to collect reference information from multiple readers for training and validation datasets in addition to the test data, especially for estimation tasks in which reader variability is expected to be high. This could reduce overfitting results to a single truther and potentially produce more generalizable algorithm performance. The advantage of utilizing multiple truthers for all data in a challenge still needs to be weighed against the time and costs associated with collecting this additional information.

## 5 Conclusion

The SPIE-AAPM-NCI BreastPathQ Challenge showed that better performing AI/ML algorithms submitted as part of the challenge were able to approach the performance of the truthing pathologist for cellularity assessment and that they may have utility in clinical practice by improving efficiency and reducing reader variability if they can be validated on larger, clinically relevant datasets. The BreastPathQ Challenge was successful because experts in multiple fields worked together on the Organizing Committee. This enabled participants to quickly understand the basics of the task, download the data, develop their algorithms, and receive efficient feedback during the training and validation phases. The BreastPathQ Challenge information is accessible on the Grand Challenge website.^41^ The data used in the challenge including the WSI scans and additional clinical information related to each patient can be found on the cancer imaging archive (TCIA).^42^

## 6 Appendix A: PK Performance by Team

Table of the best average PK results and corresponding ICC scores for each participating team along with the teams’ members, affiliations, and a brief description of their submitted algorithm.

## 7 Appendix B: BreastPathQ Challenge Group Members

List of the BreastPathQ Challenge Group members considered as co-authors on this manuscript.

**Table 2:** List of registered teams who submitted a valid test submission to BreastPathQ, a brief summary of the team’s submitted algorithms along with their best performing average PK and corresponding average ICC scores. Note that teams were allowed to submit up to three algorithms in the BreastPathQ test phase.

Rank	Team	Affiliation	Learning architecture	Optimizer	Epochs	Postprocessing	PK scores	ICC scores
1	dchambers	Southwest Research Institute; University of Texas Health, San Antonia, Texas	Inception with squeeze-excitation	Adam	25	Retrieve predictions for four 90 deg rotations and average results	0.941 [0.917, 0.958]	0.934 [0.908,0.954]
2	DRF	School of Computing, Tokyo Institute of Technology, Kanagawa, Japan	ResNet50 with squeeze-excitation	Momentum	50	Flip and rotate test patches resulting in eight predictions per patch before averaging	0.941 [0.920,0.957]	0.938 [0.913,0.956]
3	koalaryTsinghua	AI Center, Research Institute of Tsinghua, Pearl River Delta, China	Ensemble of Xception, Inception and InceptionResNet	Adam	200	Concatenate second-last layer in each network for resulting output prediction	0.939 [0.911,0.958]	0.936 [0.906,0.957]
4	FOP	Department of Computer Science, School of Information Science and Engineering, Xiamen University, Xiamen, China	DenseNet	SGD	100	The subtraction of two outputs from the 111th layer of DenseNet used to determine input image with highest score.	0.937 [0.916,0.955]	0.922 [0.892,0.943]
6	MCPRL	Beijing University of Posts and Telecommunications, singularity.ai	ResNet50	Adam	180	ResNet output layer modified for regression output	0.934 [0.906,0.953]	0.928 [0.896,0.952]
8	Silvers	—	Ensemble of 2 DenseNet architectures	Adam	30 and 20	Averaged predictions from 10 DenseNet models (one from each fivefold) and combined with DenseNet with classification output score	0.932 [0.908,0.952]	0.911 [0.879,0.934]
9	Skychain	Skychain Inc, Moscow, Russia	InceptionResNet	Adam	n.p.	Concatenate output from five InceptionResNets (fivefold) and appended regression layer	0.932 [0.908,0.951]	0.931 [0.901,0.954]
11	ice_code	IIP Lab, University of Science and Technology of China (USTC), China	Custom network containing two Xception networks; for segmentation and cellularity scoring	Adam	80	Concatenate output from multiple models (see description) and appended regression layer	0.927 [0.908,0.947]	0.861 [0.807,0.901]
14	PVmed	PVmed Inc., Guangzhou, China	InceptionResNet	Adam	100	InceptionResNet output layer modified for regression output	0.926 [0.902,0.942]	0.895 [0.852,0.924]
18	hels	Computer-Aided Diagnosis Laboratory, University of Oklahoma, Oklahoma	ResNet with and without Squeeze-Excitation layers	Adam	195 and 323	An ensemble of three models trained under different configurations.	0.923 [0.890,0.950]	0.908 [0.845,0.949]
20	SCI	Dept. of Electrical and Computer Engineering, University of Utah, Utah	ResNet	Momentum	50	Combined regression, classification, and cell counts scores. Predictions were made of multiple rotated patches and then averaged	0.923 [0.904,0.938]	0.904 [0.874,0.930]
26	SRIBD	Shenzhen Research Institute of Big Data, Shenzhen, China	Ensemble of ResNet, SENet, InceptionResNet	Adam	150	Used a Gaussian smoothing function to distribute discrete labels into neighboring labels	0.919 [0.893,0.943]	0.910 [0.873,0.939]
27	tirdad	Department of Computer Science, Ryerson University, Toronto, Canada	Inception	n.p.	200	Inception output layer is modified for regression output	0.919 [0.890,0.943]	0.885 [0.835,0.918]
29	Hanse	Fraunhofer MEVIS, Bremen, Germany; Fraunhofer MEVIS, Lübeck, Germany; Community Practice for Pathology, Lübeck, Germany	Xception, Inception	Adam	200	Also trained a UNet to segment tumor regions that was appended as a separated channel to networks. Predictions from each network were averaged.	0.918 [0.899,0.934]	0.902 [0.870,0.926]
30	TK	National University of Sciences and Technology, H-12, Islamabad, Pakistan	VGG	n.p.	25	VGG output layer modified for regression output	0.917 [0.891,0.939]	0.900 [0.859,0.930]
31	FDU-MIA	School of Computer Science and Technology, Fudan University	2 DenseNets trained on RGB and HSV color spaces	n.p.	200	Concatenation of two DenseNets and another regression layer	0.917 [0.887,0.940]	0.902 [0.863,0.929]
33	max0r	Leibniz Universität Hannover, Institute of Mechatronic Systems, Hannover, Germany	Regressive adversarial autoencoder (rAAE) containing Inception	Adam	200	Average of three rAAEs	0.916 [0.895,0.933]	0.906 [0.877,0.928]
36	VIPLabUW	Systems Design Engineering, University of Waterloo, Canada; Department of Engineering, University of Guelph, Canada; Vector Institute, Canada; Waterloo Artificial Intelligence Institute, Canada	ResNet	Adam	40	Greedy ensembling of nine ResNet models	0.916 [0.896,0.932]	0.855 [0.810,0.888]
37	HUSTyixuan	School of Electronic Information and Communications, Huazhong University of Science and Technology (HUST), Wuhan, China	Fusion-net with Deeplab V3+ and Resnet101	SGD and Adam	10,000 and 900	Output of a segmentation network used as the input for a Resnet-101 network with regression output	0.915 [0.897,0.932]	0.904 [0.880,0.926]
38	TensorMONK	University of Missouri–Kansas City, Kansas City	An autoencoder segmentation network with ResNet-18	Adam	10,000 iterations	Output of the segmentation network used as the input for a ResNet-18 network with regression output	0.915 [0.890,0.936]	0.904 [0.874,0.929]
40	UTD-QBIL	Department of Bioengineering, University of Texas; Department of Biomedical Engineering, Georgia Institute of Technology, and Emory University, Atlanta; Advanced Imaging Research Center, University of Texas Southwestern Medical Center, Texas; Department of Radiology, University of Texas Southwestern Medical Center, Texas	Inception and custom cascade network	Adadelta	300	Average of 2 CNNs	0.913 [0.893,0.928]	0.906 [0.874,0.929]
41	AVSASVA	—	ResNet-18	SGD	100	ImageNet pretrained ResNet-18 feature extractor into XGBoost	0.913 [0.892,0.931]	0.914 [0.884,0.937]
45	rakhlin	Neuromation, Tallinn, Estonia	U-Net with ResNet and Gradient Boosted Trees	n.p.	n.p.	U-Net using ResNet-34 encoder segmented the cell nuclei, then ResNet-34 or a Gradient Boosted Trees for regression	0.912 [0.892,0.929]	0.903 [0.872,0.928]
53	Bio-totem & SYSUCC	Bio-totem Pte Ltd., Foshan, China; Sun Yat-sen University Cancer Center, Guangzhou, China	ResNet-50, Mask R-CNN with ResNet-101, Xception	Adam, SGD	40, 20, 30	ResNet-50 to classify normal and tumor patches, then Mask R-CNN to segment the nuclei, then Xception network to classify the nuclei as normal or malignant. Heatmap of malignant nuclei used to calculate cellularity.	0.907 [0.861,0.939]	0.890 [0.821,0.934]
55	Boston Meditech Group	Boston Meditech Group, Boston, Massachusetts	Inception_Resnet_v2	RMSPROP	260	Inception_Resnet_v2 modified for regression task with mean absolute error (MAE) as error function.	0.904 [0.872,0.930]	0.895 [0.836,0.931]
56	IMAGe	Medical Image Analysis Group, Department of Biomedical Engineering, Eindhoven University of Technology, Eindhoven, The Netherlands	Custom CNN, Custom CNN with nuclei mask, U-Net, Ranking model	n.p.	n.p.	For the ranking model, trained a network that uses a bubble sort algorithm to compare which of two pairs of patches had higher cellularity.	0.902 [0.877,0.930]	0.876 [0.839,0.906]
57	hbk	School of Biomedical Engineering, Southern Medical University, Guangzhou, China	VGG, ResNet-50, Inception-v3 as feature extractors	SGD	n.p.	Uses pretrained three networks as feature extractor, then used gradient boosted regression model and a pairwise ranking model.	0.900 [0.879,0.921]	0.888 [0.851,0.919]
66	MeghanaKranthi	National Institute of Technology Warangal, India	ResNet-34	SGD	25	Regression with ResNet-34, with test-time data augmentation averaging the scores from the 8 rotation transform outputs.	0.880 [0.838,0.910]	0.579 [0.540,0.611]
67	Pace	Southern CT State University, New Haven, Connecticut	Xception for feature extractor	n.a.	n.a.	DataRobot AutoML platform used using fivefold cross-validation. The model produced ENET Blender, Advanced AVG Blender, and Nystroem Kernal SVM Regressor.	0.879 [0.856,0.900]	0.858 [0.810,0.902]
68	RHIT	Rose-Hulman Institute of Technology, Terre Haute, Indiana	VGG-16	Adam	n.p.	Pretrained VGG-16 with regression output.	0.876 [0.848,0.899]	0.859 [0.802,0.897]
70	MIG CDS IISC	Medical Imaging Group, Department of Computational and Data Sciences, Indian Institute of Science, Bangalore, India	Custom network	Adam	400	Training performed in four phases, with weights corresponding to lowest validation loss saved in each phase. Each subsequent phase retrained the model saved in the previous phase with new cases.	0.870 [0.843,0.889]	0.828 [0.779,0.865]
71	Grace	Department of Computer Science, Seidenberg School of CSIS, Pace University, New York City, New York	VGG-16 with convolutional pose machines	n.p	100	Use VGG-16 and convolutional pose segmentation to segment the cells, then calculate the percent pixel area.	0.869 [0.813,0.913]	0.812 [0.689,0.898]
72	this Should Be Optional	Computer Engineering Department, Ferdowsi University of Mashhad, Mashhad, Iran	Custom network	Adam	100	Pretrained discriminator of a GAN for generating images similar to the training data set.	0.868 [0.826,0.902]	0.837 [0.759,0.886]
73	MAK	Korea University, Seoul, South Korea	ResNet-34	Adam	25	Transfer learning using ResNet-34, with training with three different resolution with rotation/flip data augmentation.	0.866 [0.845,0.888]	0.843 [0.789,0.885]
75	Winterfell	Healthcare Technology Innovation Centre, IITM, India; Indian Institute of Technology Madras (IITM), India	Ensemble of ResNet-18, 34, 50, 101, and 152	Adam	200	A fusion network that combines the individual results is trained separately.	0.864 [0.837,0.889]	0.824 [0.780,0.871]
78	Chenchen_Tian	School of Information Science and Technology, University of Science and Technology of China, Hefei, China	Custom	SGD	70	Used MAE for loss function.	0.860 [0.777,0.908]	0.772 [0.579,0.884]
80	medVision	Vision Lab, Electrical & Computer Engineering, Old Dominion University, Virginia	Custom	Adam	300	CNN for malignant nuclei segmentation and initial tumor cellularly calculation, then fine tune with a second CNN	0.847 [0.790,0.900]	0.801 [0.709,0.872]
84	RBEI Healthcare	Robert Bosch Engineering and Business Solution, Bangalore, India	n.a.	n.a.	n.a.	Cell nuclei were segmented, then features were extracted to perform nuclei classification. A polynomial regressor with tissue architectural and spatial analysis results and the nuclei classification is used.	0.785 [0.711,0.864]	0.656 [0.497,0.796]
87	huangch	—	n.a.	n.a.	n.a.	QuPath for nucleus detection and segmentation, then an exclusive autoencoder to calculate the initial cellularity, probability of lymphocyte, probability of malignant cell, probability normal cell, then combined using a FCN.	0.497 [0.476, 0.517]	−0.012 [−0.054, 0.041]

n.p., not provided; n.a., not applicable.

**Table 3:** List of BreastPathQ Challenge group members considered to be coauthors in this paper. The table is in alphabetical order separated by challenge organizers, pathologists, and participants.

Name	Institution	Role	Team	Disclosures
Shazia Akbar	University of Toronto, Sunnybrook Research Institute, Toronto, Canada	Organizer	—	None
Kenny H. Cha	U.S Food and Drug Administration, Center for Device and Radiological Health, Silver Spring, Maryland 20993	Organizer	—	None
Diane Cline	SPIE, Bellingham, Washington	Organizer	—	—
Karen Drukker	Department of Radiology, University of Chicago, Chicago, Illinois	Organizer	—	Royalties from Hologic, Inc. (Marlborough, Massachusetts)
Keyvan F. Farahani	National Cancer Institute, National Institutes of Health, Rockville, Maryland	Organizer	—	None
Marios A. Gavrielides	U.S Food and Drug Administration, Center for Device and Radiological Health, Silver Spring, Maryland	Organizer	—	None
Lubomir M. Hadjiiski	University of Michigan, Ann Arbor, Michigan	Organizer	—	—
Jayashree Kalpathy-Cramer	Massachusetts General Hospital, Harvard University, Boston, Massachusetts	Organizer	—	Grant from GE Healthcare; Leidos contract HHSN2612008000001E
Elizabeth A. Krupinski	Emory University, Atlanta, Georgia	Organizer	—	—
Anne L. Martel	University of Toronto, Medical Biophysics, Sunnybrook Research Institute, Toronto, Canada	Organizer	—	Co-founder and CEO of Pathcore (Toronto, Ontario, Canada)
Samarth Nandekar	Massachusetts General Hospital, Harvard University, Boston, Massachusetts	Organizer	—	—
Nicholas Petrick	U.S Food and Drug Administration, Center for Device and Radiological Health, Silver Spring, Maryland	Organizer	—	None
Berkman Sahiner	U.S Food and Drug Administration, Center for Device and Radiological Health, Silver Spring, Maryland	Organizer	—	None
Joel Saltz	Department of Biomedical Informatics, Stony Brook University, Stony Brook, New York	Organizer	—	—
John Tomaszewski	Department of Pathology and Anatomical Sciences, Jacobs School of Medicine and Biomedical Sciences, University of Buffalo, Buffalo, New York	Organizer	—	—
Sharon Nofech-Mozes	University of Toronto, Department of Laboratory Medicine and Pathobiology, Sunnybrook Health Sciences Centre, Toronto, Canada	Pathologist		None
Sherine Salama	Sunnybrook Research Institute, Toronto, Ontario Canada.	Pathologist	—	—
Hassan Ali	National University of Sciences and Technology, H-12, Islamabad, Pakistan	Participant	TK	—
Cory Austin	Department of Computer Science, Ryerson University, Toronto, Ontario, Canada	Participant	tirdad	—
Navchetan Awasthi	Medical Imaging Group, Department of Computational and Data Sciences, Indian Institute of Science, Bangalore, India	Participant	MIG CDS IISC	None
Jacob Beckmann	Rose-Hulman Institute of Technology, Terre Haute, Indiana,	Participant	RHIT	—
Brad Brimhall	University of Texas Health, San Antonio, Texas	Participant	dchambers	None
Zhuoqun Cao	School of Computer Science and Technology, Fudan University, China	Participant	FDUMIA	—
ZhiZhong Chai	Department of Computer Science, School of Information Science and Engineering, Xiamen University, Xiamen, China	Participant	FOP	—
David Chambers	Southwest Research Institute, San Antonio, Texas	Participant	dchambers	None
Rongzhen Chen	Department of Computer Science, School of Information Science and Engineering, Xiamen University, Xiamen, China	Participant	FOP	—
Yihao Chen	Shenzhen Research Institute of Big Data, Shenzhen, China	Participant	SRIBD	—
Jaegul Choo	KAIST, Daejeon, South Korea	Participant	MAK	None
Alex Dela Cruz	Department of Computer Science, Ryerson University, Toronto, Ontario, Canada	Participant	tirdad	—
Ibrahim Ben Daya	Systems Design Engineering, University of Waterloo, Ontario, Canada; Waterloo Artificial Intelligence Institute, Waterloo, Ontario, Canada	Participant	VIPLab	None
Jason Deglint	Systems Design Engineering, University of Waterloo, Ontario, Canada; Waterloo Artificial Intelligence Institute, Waterloo, Ontario, Canada	Participant	VIPLab	None
James Dormer	Department of Bioengineering, University of Texas at Dallas, Texas	Participant	UTD-QBIL	—
Jingwei Du	Department of Computer Science, Seidenberg School of CSIS, Pace University, New York City, New York	Participant	Grace	—
Koen A.J. Eppenhof	Medical Image Analysis Group, Department of Biomedical Engineering, Eindhoven University of Technology, Eindhoven, The Netherlands	Participant	IMAGe	—
Jacob F. Fast	Leibniz Universität Hannover, Institute of Mechatronic Systems, Hannover, Germany	Participant	max0r	—
Baowei Fei	Department of Bioengineering, University of Texas at Dallas, Texas; Advanced Imaging Research Center, University of Texas Southwestern Medical Center, Dallas, Texas; Department of Radiology, University of Texas Southwestern Medical Center, Dallas, Texas	Participant	UTD-QBIL	—
Navid Ghassemi	Computer Engineering Department, Ferdowsi University of Mashhad, Mashhad, Iran	Participant	thisShouldBeOptional	None
Vikas Gottemukkula	—	Participant	TensorMONK	—
Limei Guo	Department of Pathology, School of Basic Medical Sciences, Third Hospital, Peking University Health Science Center, China	Participant	Huang	None
Kranthi Kiran GV	National Institute of Technology, Warangal, India	Participant	MeghanaKranthi	—
Martin Halicek	Department of Bioengineering, University of Texas at Dallas, Texas; Department of Biomedical Engineering, Georgia Institute of Technology and Emory University, Atlanta, Georgia	Participant	UTD-QBIL	—
Lanqing Han	AI Center, Research Institute of Tsinghua, Pearl River Delta, China	Participant	koalaryTsinghua	None
Linsheng He	Computer-Aided Diagnosis Laboratory, University of Oklahoma, Oklahoma	Participant	hels	—
Yifan He	Pvmed Inc., Guangzhou, China	Participant	PVmed	—
Friso G. Heslinga	Medical Image Analysis Group, Department of Biomedical Engineering, Eindhoven University of Technology, Eindhoven, The Netherlands	Participant	IMAGe	—
Henning Höfener	Fraunhofer MEVIS, Bremen, German	Participant	Hanse	None
Wang Huajia	Bio-totem Pte Ltd., Foshan, China	Participant	Bio-totem & SYSUCC	—
Chao-Hui Huang	—	Participant	huangch	None
Guifang Huang	AI Center, Research Institute of Tsinghua, Pearl River Delta, China	Participant	koalaryTsinghua	None
Hui Hui	Bio-totem Pte Ltd., Foshan, China	Participant	Bio-totem & SYSUCC	—
Khan M. Iftekharuddin	Vision Lab, Electrical and Computer Engineering, Old Dominion University, Norfolk, Virginia	Participant	medVision	—
Humayun Irshad	Boston Meditech Group, Boston, Massachusetts	Participant	Boston Meditech Group	—
Yang Jiahua	Bio-totem Pte Ltd., Foshan, China	Participant	Bio-totem & SYSUCC	—
Longquan Jiang	School of Computer Science and Technology, Fudan University, China	Participant	FDUMIA	—
Kuang Jinbo	Bio-totem Pte Ltd., Foshan, China	Participant	Bio-totem & SYSUCC	—
Lüder A. Kahrs	Leibniz Universität Hannover, Institute of Mechatronic Systems, Hannover, Germany	Participant	max0r	—
Ananth Kalyanasundaram	Healthcare Technology Innovation Centre, IITM, India	Participant	Winterfell	—
Mohammad A. Khan	Korea University, Seongbuk-gu, Seoul, South Korea	Participant	MAK	None
Devinder Kumar	Systems Design Engineering, University of Waterloo, Ontario, Canada; Vector Institute for AI, Toronto, Ontario, Canada; Waterloo Artificial Intelligence Institute, Waterloo, Ontario, Canada	Participant	VIPLabUW	None
Max-Heinrich Laves	Leibniz Universität Hannover, Institute of Mechatronic Systems, Hannover, Germany	Participant	max0r	—
Ao Li	School of Information Science and Technology, University of Science and Technology of China, Hefei, China	Participant	Chenchen_Tian	—
Liu Li	School of Computer Science and Technology, Fudan University, China	Participant	FDUMIA	—
Quan Li	AI Center, Research Institute of Tsinghua, Pearl River Delta, China	Participant	koalaryTsinghua	None
Ziqiang Li	IIP Lab, USTC, China	Participant	ice_code	—
Yu Liu	School of Information Science and Technology, University of Science and Technology of China, Hefei, China	Participant	Chenchen_Tian	—
Johannes Lotz	Fraunhofer MEVIS, Lübeck, Germany	Participant	Hanse	None
Cuong Ly	Department of Electrical and Computer Engineering, University of Utah, Salt Lake City, Utah	Participant	SCI	—
Zhu Meng	Beijing University of Posts and Telecommunications, Singularity.ai Beijing, China	Participant	MCPRL	None
Balamurali Murugesan	Healthcare Technology Innovation Centre, IITM, India; Indian Institute of Technology Madras, India	Participant	Winterfell	—
Dmitry Musinov	Skychain Inc., Moscow, Russia	Participant	Skychain	—
Mamada Naoya	School of Computing, Tokyo Institute of Technology, 4259, Nagatsuta Midori-ku, Yokohama-shi, Kanagawa, Japan	Participant	nattochaduke	—
Wajahat Nawaz	National University of Sciences and Technology, H-12, Islamabad, Pakistan	Participant	TK	—
Anusha Nayak	Robert Bosch Engineering and Business Solution, Bangalore, India	Participant	RBEI Healthcare	—
Sergey Nikolenko	Neuromation, Tallinn, Estonia	Participant	rakhlin	—
Hongjing Niu	IIP Lab, University of Science and Technology of China (USTC), China	Participant	ice_code	—
Tobias Ortmaier	Leibniz Universität Hannover, Institute of Mechatronic Systems, Hannover, Germany	Participant	max0r	—
Rohit Pardasani	Medical Imaging Group, Department of Computational and Data Sciences, Indian Institute of Science, Bangalore, India	Participant	MIG CDS IISC	None
Linmin Pei	Vision Lab, Electrical and Computer Engineering, Old Dominion University, Norfolk, Virginia	Participant	medVision	—
Ziang Pei	School of Biomedical Engineering, Southern Medical University, Guangzhou, China	Participant	hbk	—
Sun Peng	Sun Yat-sen University Cancer Center, Guangzhou, China	Participant	Bio-totem & SYSUCC	—
Josien P.W. Pluim	Medical Image Analysis Group, Department of Biomedical Engineering, Eindhoven University of Technology, Eindhoven, The Netherlands	Participant	IMAGe	—
Kosta Popovic	Rose-Hulman Institute of Technology, Terre Haute, Indiana	Participant	RHIT	—
Xianbiao Qi	Shenzhen Research Institute of Big Data, Shenzhen, China	Participant	SRIBD	—
Vikrant Raghu	Robert Bosch Engineering and Business Solution, Bangalore, India	Participant	RBEI Healthcare	—
Alexander Rakhlin	Neuromation, Tallinn, Estonia	Participant	rakhlin	—
Keerthi Ram	Healthcare Technology Innovation Centre, IITM, India	Participant	Winterfell	—
G Meghana Reddy	National Institute of Technology, Warangal, India	Participant	MeghanaKranthi	—
Yong Ren	AI Center, Research Institute of Tsinghua, Peral River Delta, China	Participant	koalaryTsinghua	None
Anthony S. Richardson	Southern CT State University, New Haven, Connecticut	Participant	Pace	—
Modjtaba Rouhani	Computer Engineering Department, Ferdowsi University of Mashhad, Mashhad, Iran	Participant	thisShouldBeOptional	None
Alireza Sadeghian	Department of Computer Science, Ryerson University, Toronto, ON, Canada	Participant	Tirdad	—
Sashi Saripalle	—	Participant	TensorMONK	—
Kaushik Sarveswaran	Healthcare Technology Innovation Centre, IITM, India	Participant	Winterfell	None
Lars Ole Schwen	Fraunhofer MEVIS, Bremen, Germany	Participant	Hanse	—
Maysam Shahedi	Department of Bioengineering, University of Texas at Dallas, Texas	Participant	UTD-QBIL	—
Subbashini Shanmugam	Robert Bosch Engineering and Business Solution, Bangalore, India	Participant	RBEI Healthcare	—
Afshin Shoeibi	Computer Engineering Department, Ferdowsi University of Mashhad, Mashhad, Iran	Participant	thisShouldBeOptional	None
Xu Shuoyu	Bio-totem Pte Ltd., Foshan, China; Sun Yat-sen University Cancer Center, Guangzhou, China	Participant	Bio-totem&SYSUCC	—
Mohanasankar Sivaprakasam	Healthcare Technology Innovation Centre, IITM, India; Indian Institute of Technology Madras, India	Participant	Winterfell	—
Louisa Spahl	Fraunhofer MEVIS, Lübeck, Germany	Participant	Hanse	None
Fei Su	Beijing University of Posts and Telecommunications, Singularity.ai Beijing, China	Participant	MCPRL	None
Lei Su	School of Information Science and Technology, University of Science and Technology of China, Hefei, China	Participant	Chenchen_Tian	—
Tolga Tasdizen	Department of Electrical and Computer Engineering, University of Utah, Salt Lake City, Utah	Participant	SCI	—
Graham W. Taylor	Department of Engineering, University of Guelph, Ontario, Canada; Vector Institute for AI, Toronto, Ontario, Canada	Participant	VIPlabUW	None
Chenchen Tian	School of Information Science and Technology, University of Science and Technology of China, Hefei, China	Participant	Chenchen_Tian	—
Kayvan Tirdad	Department of Computer Science, Ryerson University, Toronto, Ontario, Canada	Participant	Tirdad	—
Andreas Turzynski	Community practice for pathology Lübeck, Lübeck, Germany	Participant	Hanse	None
Krishnan Venkataraman	Robert Bosch Engineering and Business Solution, Bangalore, India	Participant	RBEI Healthcare	—
Mitko Veta	Medical Image Analysis Group, Department of Biomedical Engineering, Eindhoven University of Technology, Eindhoven, The Netherlands	Participant	IMAGe	—
Liansheng Wang	Department of Computer Science, School of Information Science and Engineering, Xiamen University, Xiamen, China	Participant	FOP	—
Shuxin Wang	Department of Computer Science, School of Information Science and Engineering, Xiamen University, Xiamen, China	Participant	FOP	—
Yixuan Wang	School of Electronic Information and Communications, Huazhong University of Science and Technology (HUST), Wuhan, China	Participant	HUSTyixuan	—
Nick Weiss	Fraunhofer MEVIS, Lübeck, Germany	Participant	Hanse	None
Suzanne C. Wetstein	Medical Image Analysis Group, Department of Biomedical Engineering, Eindhoven University of Technology, Eindhoven, The Netherlands	Participant	IMAGe	—
Alexander Wong	Systems Design Engineering, University of Waterloo, Waterloo, ON, Canada; Waterloo Artificial Intelligence Institute, Waterloo, Ontario, Canada	Participant	VIPlabUW	None
Zihan Wu	Department of Computer Science, School of Information Science and Engineering, Xiamen University, Xiamen, China	Participant	FOP	—
Zihan Wu	—	Participant	Silvers	—
Xiaodong Xu	Department of Computer Science, Seidenberg School of CSIS, Pace University, New York City, New York	Participant	Grace	—
Songlin Yang	Department of Computer Science, School of Information Science and Engineering, Xiamen University, Xiamen, China	Participant	FOP	—
Junjie Ye	AI Center, Research Institute of Tsinghua, Peral River Delta, China	Participant	koalaryTsinghua	None
Li Yu	School of Electronic Information and Communications, Huazhong University of Science and Technology (HUST), Wuhan, China	Participant	HUSTyixuan	—
Ding Yuguo	Boston Meditech Group, Boston, Massachusetts	Participant	Boston Meditech Group	—
Zhicheng Zhao	Beijing University of Posts and Telecommunications, Singularity.ai Beijing, China	Participant	MCPRL	None
Yun Zhu	School of Computer Science and Technology, Fudan University, China	Participant	FDUMIA	—
Andrey Zhylka	Medical Image Analysis Group, Department of Biomedical Engineering, Eindhoven University of Technology, Eindhoven, The Netherlands	Participant	IMAGe	—
—	—	Participant	AVSASVA	—
